# Two Cycloartenol Synthases for Phytosterol Biosynthesis in *Polygala tenuifolia* Willd

**DOI:** 10.3390/ijms18112426

**Published:** 2017-11-15

**Authors:** Mei Lan Jin, Woo Moon Lee, Ok Tae Kim

**Affiliations:** Department of Herbal Crop Research, National Institute of Horticultural and Herbal Science, Rural Development Administration, Eumseong 27709, Korea; milan80623@korea.kr (M.L.J.); wmlee65@korea.kr (W.M.L.)

**Keywords:** cycloartenol synthase, oxidosqualene cyclase, phytosterols, triterpenoids, *Polygala tenuifolia*

## Abstract

Oxidosqualene cyclases (OSCs) are enzymes that play a key role in control of the biosynthesis of phytosterols and triterpene saponins. In order to uncover *OSC* genes from *Polygala tenuifolia* seedlings induced by methyl jasmonate (MeJA), RNA-sequencing analysis was performed using the Illumina sequencing platform. A total of 148,488,632 high-quality reads from two samples (control and the MeJA treated) were generated. We screened genes related to phytosterol and triterpene saponin biosynthesis and analyzed the transcriptional changes of differentially expressed unigene (DEUG) values calculated by fragments per kilobase million (FPKM). In our datasets, two full-length cDNAs of putative *OSC* genes, *PtCAS1*, and *PtCAS2*, were found, in addition to the *PtBS* (β-amyrin synthase) gene reported in our previous studies and the two cycloartenol synthase genes of *P. tenuifolia*. All genes were isolated and characterized in yeast cells. The functional expression of the two *PtCAS* genes in yeast cells showed that the genes all produce a cycloartenol as the sole product. When qRT-PCR analysis from different tissues was performed, the expressions of *PtCAS1* and *PtCAS2* were highest in flowers and roots, respectively. After MeJA treatment, the transcripts of *PtCAS1* and *PtCAS2* genes increased by 1.5- and 2-fold, respectively. Given these results, we discuss the potential roles of the two *PtCAS* genes in relation to triterpenoid biosynthesis.

## 1. Introduction

**Figure 1:**
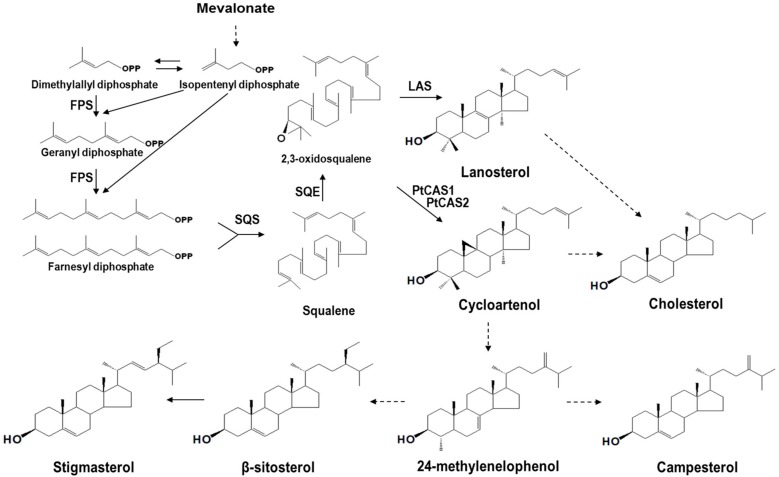
Phytosterol biosynthesis in *P. tenuifolia*. FPS, farnesyl diphosphate synthase; SQS, squalene synthase; SQE, squalene epoxidase; LAS, lasnosterol synthase. Dashed lines indicate multi-steps.

The cyclization of 2,3-oxidosqualene, which is catalyzed by several oxidosqualene cyclases (OSCs) results in the formation of various triterpenoids. These processes are completed through the protonation of epoxide, rearrangement of carbocation, and termination by deprotonation [1]. A protosteryl cation can convert 2,3-oxidosqualene to lanosterol, cycloartenol, and curcurbitadinol of chair-boat-chair type series reactions; and a dammarenyl cation can convert lupeol, β-amyrin, taraxerol, and other triterpenoids of chair-chair-chair type series reactions [2]. Cycloartenol synthase (CAS, EC 5.4.99.8) catalyzes the cyclization of 2,3-oxidosqualene and finally produces a cycloartenol. Phytosterols, which are biosynthesized through oxidation and hydroxylation, are an essential element for forming plasma membranes. The most common sterols that accumulate in plants are β-sitosterol, stigmasterol, and campesterol (Figure 1). Recently, it has been reported that the CAS protein plays a vital role in sterol biosynthesis, which is essential for plant cell viability [3]. Consistent with this, the *cas1* mutant lines of *Arabidopsis* exhibit lower levels of sterols than the control. Only one *CAS* (cycloartenol synthase) gene exists in the *Arabidopsis* genome [4], whereas two *CAS* genes, *BPX* and *BPX2*, from the *Butula platyphylla* were cloned and characterized in yeast cells [5]. Thus far, any advanced studies on the different functions between the *CAS* genes of a plant have not yet been reported.

*Polygala tenuifolia*, which is a well-known traditional Asian medicine for treating phlegm and for detumescence, is a perennial herb widely distributed in the north of China and Korea. In China, triterpene saponins, which are derived from this herb, were one of the principal components of some medicines, given their chemical and pharmacological properties [6]. *P. tenuifolia* contains 34 different oleanane-type triterpene saponins, belonging to the groups of onjisaponins and polygalasaponins [7]. However, to date, steroidal saponins as secondary metabolites have not been identified in the plant.

To uncover *OSC* genes of the *P. tenuifolia*, we employed the Illumina sequencing platform for transcriptomic analysis of 5-week old seedlings induced by methyl jasmonate (MeJA), which is a signaling molecule involved in the regulation of defense mechanisms against fungal pathogens, environmental stress, and injury [8,9]. In particular, MeJA can stimulate not only the production of secondary metabolites such as sesquiterpenoids and triterpenoids but also the high expression of genes associated with pathways involved in cell suspension, hairy root, and adventitious root cultures (e.g., β-amyrin synthase (bAS), cytochrome P450 (CYP), and glucosyltransferase (UGT)). Recently, Tian et al. [10] reported the results of a transcriptome analysis of wild *P. tenuifolia* roots. In their study, a total of 70 unigenes of OSCs (16 for a CAS and 54 for a bAS) were discovered from the dataset, although the information on the unigenes was not described in detail.

In this study, two cDNA libraries were constructed from 5-week old (24 h) MeJA treated or untreated seedlings, in order to uncover candidate genes related to the triterpenoid pathway by using a high-throughput Illumina deep-sequencing technique. Three genes of approximately 2.3 kb in size, likely to be full-length cDNAs of *OSC*, were determined. Two putative *CAS* genes were isolated from the cDNA pool and characterized by heterologous expression in yeast mutant cells. Further, the expression of the *CAS* genes in *P. tenuifolia* was investigated by real-time RT-PCR analysis.

## 2. Results

### 2.1. De Novo Transcriptome Assembly and Functional Annotation

**Figure 2:**
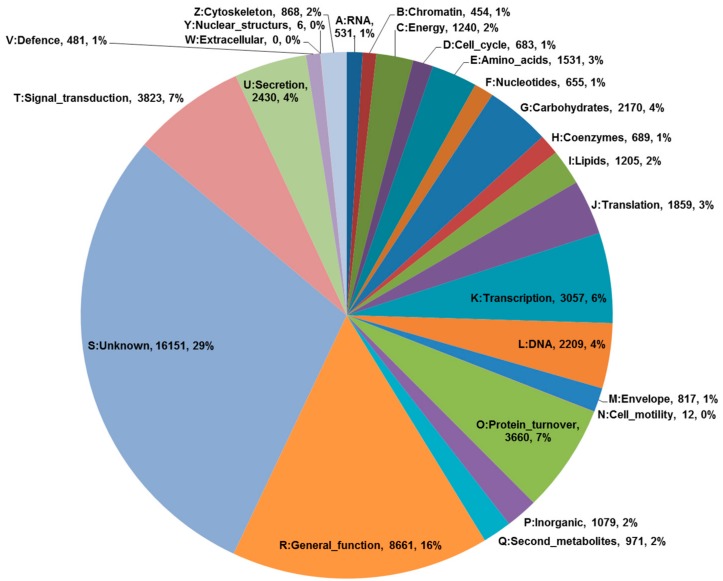
Results of an eggNOG functional category analysis of 55,242 unigenes. All identified proteins were classified into molecular families.

To generate the transcriptome of *P. tenuifolia*, total RNAs isolated from 5-week old seedlings treated with MeJA were subjected to sequencing on an Illumina platform. A total of 148,488,632 high-quality reads were generated: 71.8 million for the control sample and 76.7 million for the MeJA treated sample (Table 1). *De novo* assembly of total high-quality reads resulted in the generation of 129,035 unigenes with a GC content of about 45.3%, where the average length of each unigene was 1065 bp. A total of 129,035 unigenes were expressed, with the greatest abundance observed with the sample treated with MeJA. Out of the expressed unigenes, a total of 54,931 (42.6%), 69,060 (53.5%), and 66,649 (55.5%) were identified by subjecting them to the NR (NCBI non-redundant protein), TAIR (The Arabidopsis Information Resource) and COG (Clusters of Orthologous Groups) databases, respectively. To understand the characteristics of the functional distribution of genes in *P. tenuifolia*, we used eggNOG annotation [11], which is based on the COG database. A total of 55,242 unigenes were classified into 25 groups according to their possible functions (Figure 2). Three categories predominated the unigenes: “General function”, “Signal transduction”, and “Protein turnover” (16%, 7%, and 7%, respectively). In contrast, “Extracellular” and “Nuclear structures” showed the least correlation.

### 2.2. Simple Sequence Repeats (SSRs) in the P. tenuifolia Transcriptome

In this work, a large number of SSRs were identified in the transcriptome data. Based on the motifs, all SSRs were divided into repeats: from di- to hexa-nucleotide repeats (Table 2). Out of the repeat motifs in the *P. tenuifolia* transcriptome, tri-nucleotide repeats were the most abundant (1208, 61.9%), followed by di-nucleotide (574, 29.4%), tetra-nucleotide (112, 5.7%), hexa-nucleotide (31, 1.6%), and penta-nucleotide (25, 1.3%). These SSR subsets can be used extensively for linkage map development, marker assisted selection, genetic diversity studies, and gene flow and environmental studies [12,13]. In particular, transcript-based molecular markers can be considered an effective tool for classifying functional genetic variations. Nevertheless, SSRs from the *P. tenuifolia* transcriptome have not been reported until now.

### 2.3. Identification of Differential Gene Expression

To investigate the differential gene expression in response to MeJA, a total of 125,012 differentially expressed unigenes (DEUGs) were analyzed (Table S1). A dramatic increase of expression of numerous genes was observed in response to MeJA. Among the abundant transcripts (as compared to the control), several well-known transcription factors such as ethylene response factor (ERF), WRKY transcription factor, etc. [9] ranked high. Moreover, we found 57 unigenes related to phytostrol biosynthesis (Table S2).

The unigenes encoding enzymes involved in triterpenoid biosynthesis, including squalene synthase (SQS, EC 2.5.1.21), squalene epoxidase (SQE, EC 1.14.99.7), and oxidosqualene cyclase (OSC, EC 5.4.99.8) were identified (Table 3). SQS, which catalyzes head to tail joining of two units of farnesyl diphosphate to squalene, plays a role in providing the first committed precursor for steroid and triterpene biosynthesis. The transcriptome data of *P. tenuifolia*, as determined by RNA-seq, indicated that the sequences share a 99% identity with the protein sequences of TBIU1284 of *P. tenuifolia* SQS (DQ672339), registered on the NCBI database. In the next step of biosynthesis, SQE converts squalene into 2,3-oxidosqualene. In *A. thaliana*, six *SQE* genes exist in the genome. However, their physiological functions are still unknown, except for the *SQE1* gene of *A. thaliana*, which contributes to root and seed development [14]. In our study, five unigenes for SQE were detected in the transcripotome.

**Figure 3:**
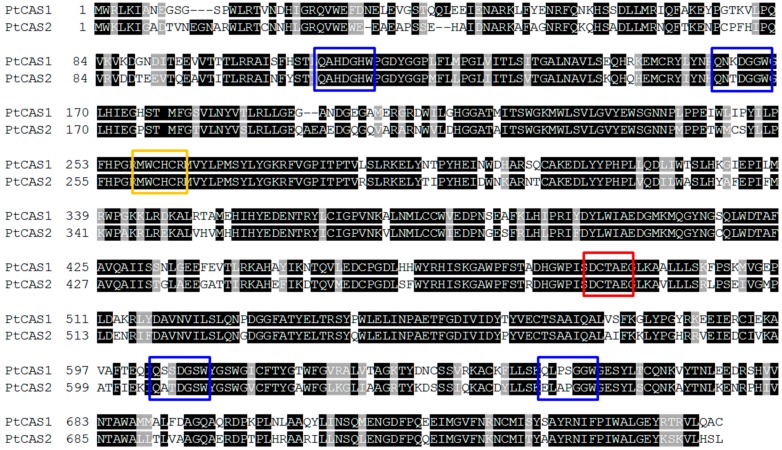
Alignment of the deduced amino acid sequences of two PtCASs from *P. tenuifolia.* Blue, yellow, and red boxes indicate the QXXXGXW motifs, the highly conserved motif among CAS genes, and the DCTAE motif, respectively.

**Figure 4:**
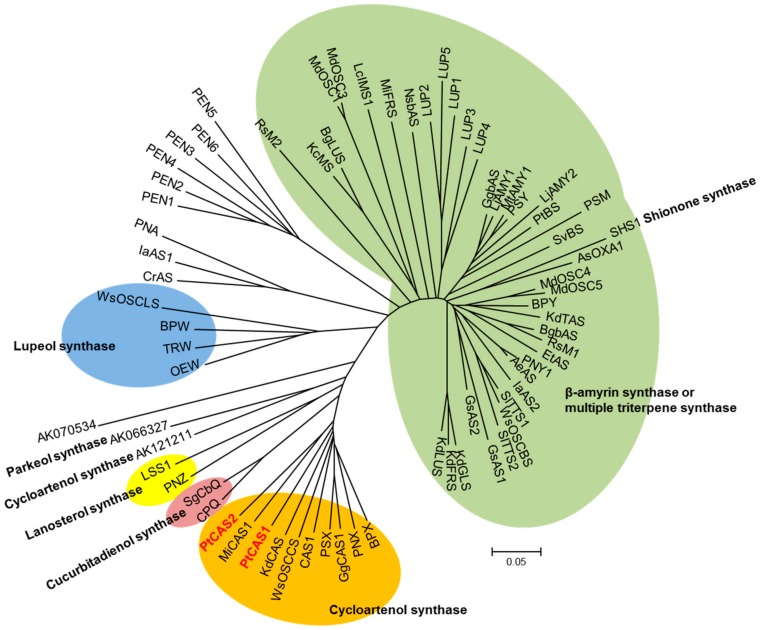
Phylogenetic tree of PtCAS1 and PtCAS2 with other plant OSCs. Gene accession numbers are given in Table S3.

Various members of the *OSC* gene family can generate numerous triterpenoids by cyclization of 2,3-oxidosqualene, which is the branch point between phytosterol and triterpenoid biosynthesis. bAS leads to the synthesis of a lot of oleanane-type triterpenoids, whereas CAS provides phytosterols with the carbon source as a precursor. In our datasets, eleven unigenes for OSC were determined; and three *bAS* and eight *CAS* unigenes were subsequently annotated. Out of those, the sequences of three unigenes for bAS were consistent with those of the *PtBS* gene (EF107623) reported by Jin et al. [15]. Eight unigenes for CAS appeared to have open reading frames (ORFs) while two unigenes possessed full-length cDNAs that were identified by comparing amino acid sequences. We named the genes *PtCAS1* and *PtCAS2* genes for CAS in *P. tenuifolia*. The PtCAS1 and PtCAS2 deduced from amino acid sequences are predicted to encode 762 and 764 amino acids with masses of 86.826 and 86.895 kDa, respectively. Both genes contain the DCTAE motif and four QXXXGXW motifs as characteristic of OSCs (Figure 3). The two genes also possessed the MWCHCR motif which *CAS* genes commonly have, but its function in triterpene cyclization is still unclear. To elucidate the phylogenetic relationship of the two PtCASs to the plant OSCs, we constructed a phylogenetic tree based on the deduced amino acid sequences. As predicted, the two genes grouped clearly with CAS (Figure 4). The two *CAS* genes in *P. tenuifoloa* were identified with the transcriptome data by RNA-seq analysis. This finding motivated us to elucidate the relationship between the two genes to phytosterol biosynthesis in *P. tenuifolia*.

### 2.4. Functional Characterization of PtCAS1 and PtCAS2 Genes in Yeast Cells

**Figure 5:**
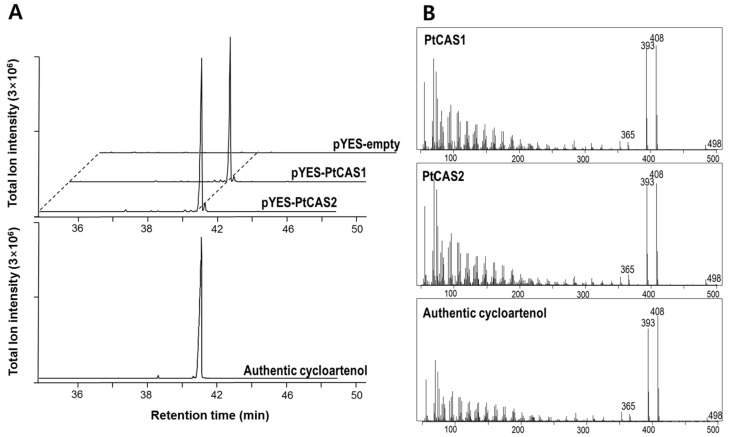
Identification of cycloartenol in yeast cells overexpressing *PtCAS1* and *PtCAS2* genes by Gas chromatography-mass spectrometry (GC-MS) analysis. (**A**) GC chromatograms of cycloartenol authentic standard, strain expressing *PtCAS1* and *PtCAS2* and control yeast strain harboring pYES2 vector; (**B**) MS spectrum of trimethylsilylated cycloartenol produced from strain expressing *PtCAS1* and *PtCAS2* and the standard cycloartenol.

We constructed vectors to express the *PtCAS1* and *PtCAS2* genes by the heterologous expression system in yeast cells. After confirmation of the cDNA sequences, two clones were finally selected and plasmids harboring each gene were used to transform into the yeast mutant GIL77 cells. As per the protocol described by Kushiro et al. [16], transgenic cells were cultured after the addition of galactose for one day. To identify the products in the extracts of the yeast cells expressing *PtCAS1* or *PtCAS2*, we conducted gas chromatography-mass spectrometry (GC-MS) analysis. As shown in Figure 5A, both PtCAS1 and PtCAS2 extracts contained one peak that was not visible with the control cells carrying the empty vector. The two peaks were identified as a cycloartenol by comparing their retention times and mass fragment patterns to the authentic standard (Figure 5B). Therefore, both *CAS* genes produced cycloartenol by catalyzing 2,3-oxidosqualene.

### 2.5. Expressions of Two CAS Genes in Organs and in Seedlings Treated with MeJA

**Figure 6:**
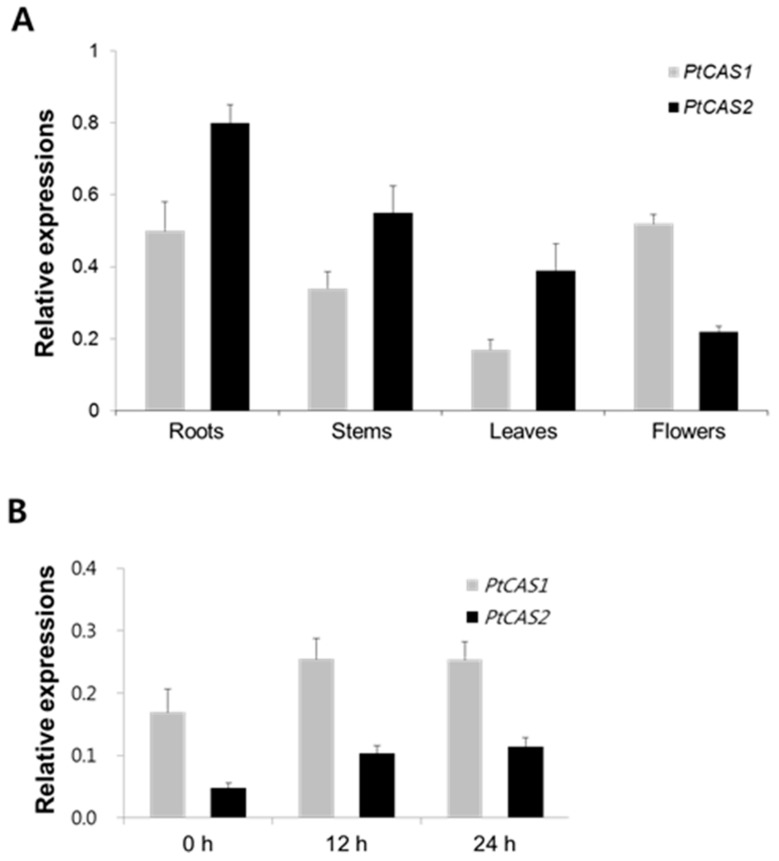
Results of qRT-PCR analysis. (**A**) Relative gene expression of *PtCAS1* and *PtCAS2* genes in organs of *P. tenuifolia* cultured in the field for 5 months and (**B**) time course after MeJA. After pre-cultivation on the ½ Murashige and Skoog (MS) medium for 5 weeks, seedlings were elicited by 0.1 mM MeJA. β-actin gene was used as the reference gene. The error bars represent standard errors from three biological replicates.

To get insight into the differences in the gene expression of the organs of *P. tenuifolia* cultured in the field for five months, qRT-PCR analysis was conducted. As shown in Figure 6A, the expression level of the *PtCAS1* gene was highest in the roots, whereas the *PtCAS2* gene was most abundant in the flowers. These results suggest that the physiological roles may differ between the two genes. The expression of specific genes such as the *bAS* gene, which leads to up-regulation of triterpenoid saponin biosynthesis, was enhanced in response to MeJA. However, the expression of the *CAS* genes was not significantly different in spite of MeJA treatment, conceivably, to support phytosterol biosynthesis [17,18]. Interestingly, the transcript patterns of the two *PtCAS* genes of the seedling of *P. tenuifolia* after MeJA treatment were similar. In the case of the two *PtCAS* genes of *P. tenuifolia*, the results of the qRT-PCR analysis showed that the transcripts of *PtCAS1* and *PtCAS2* genes increased by 1.5- and 2-fold, respectively, in response to MeJA (Figure 6B). This result corresponded to the DEUGs of the *PtCAS* genes after MeJA treatment. Similarly, Mishra et al. [19] reported that transcript levels of the *WsCAS* (*Withania somnifera* cycloartenol synthase) gene of the *W. somnifera* seedling, which contains a large amount of steroidal triterpene saponins, increased up to 1.65-fold after MeJA treatment. Taken together, these results suggest that the *PtCAS* genes may contribute to secondary steroidal triterpenoid biosynthesis as well as to phytosterols in *P. tenuifolia*.

## 3. Discussion

In order to uncover novel genes related to sterol and triterpenoid biosynthesis, RNA-seq analysis was conducted of 5-week old seedlings of *P. tenuifolia* treated with MeJA. In plants, two genes of SQS exist in the *A. thaliana* and *Panax ginseng* [20,21]. The gene expression differs by organ and location of the two SQSs. However, only one gene for SQS was identified from the transcriptome dataset. By comparing the log fold change (LFC) from the DEUGs of the two samples, the value for TBIU012384 was determined to be 3.36, indicating that this gene is highly expressed in response to MeJA. This result was similar with that of several reported studies, where the SQS gene was upregulated in cells or tissues upon induction with MeJA [21,22,23]. Five unigenes were annotated as SQE, and the LFC of the TBIU001990 unigene was determined to be the highest. Collectively, given the difference of LFC values observed following MeJA treatment, these results point to the TBIU012384 unigene of SQS and the TBIU001990 unigene of SQE as strong candidiates, which may play a role in controlling the biosynthesis of triterpenoid saponins.

CAS produces cycloartenol as a key precursor, which plays an important role in controlling the biosynthesis of phytosterol via a series of reactions that includes desaturation, hydroxylation, demethylation, etc. CAS genes of several plants have been characterized by heterologous expression in yeast cells [5,24,25]. In this work, the results of GC-MS analysis demonstrated that PtCAS1 and PtCAS2 yielded a single cycloartenol product (Figure 5). In contrast to the *CAS* gene of *Arabidopsis*, *P. tenuifolia* contains two genes for CAS in the genome. Thus far, it is not clear why some plants contain multiple copies of *CAS* gene. According to Zhang et al. [5] one of the genes may exist to safeguard the phytosterol biosynthetic pathway against any fatal mutations that may afflict the other gene. Another theory for the existence of two *CAS* genes in a plant is that one gene is there to constantly maintain phytosterol production while the other is specifically expressed in response to different environmental conditions. qRT-PCR analysis demonstrated that expressions between the two genes differed in flowers (Figure 6A). It is likely that the *PtCAS1* gene is constantly expressed to produce phytosterols and the *PtCAS2* gene is responsible for the biosynthesis of steroidal compounds to protect themselves against changing or harsh environments such as pathogens coming into contact with flowers. In addition, CAS is activated to produce steroidal triterpenoids as secondary metabolites, like withanolide [19]. After 12 h of MeJA treatment on the seedlings of *P. tenuifolia*, the transcripts of the two *CAS* genes were higher than that of the control (Figure 6B). This result might indicate that the *CAS* genes of *P. tenuifolia* also contribute to the biosynthesis of steroidal triterpenoids, although we note that steroidal triterpenoids have not yet been identified.

## 4. Materials and Methods

### 4.1. Plant Materials and RNA Extraction

Seeds of *P. tenuifolia* were cultured on ½ Murashige and Skoog (MS) medium supplemented with 3% sucrose and 0.8% agar. Five-week old seedlings were cultured on ½ MS liquid medium containing 0.1 mM MeJA for one day, and then total RNA was isolated using the RNeasy Plant kit (Qiagen, Hilden, Germany). The quality of total RNA was measured using the Agilent 2100 Bioanalyzer (Agilent Technologies, Santa Clara, CA, USA).

### 4.2. RNA Library Preparation and Sequencing

RNA-Seq libraries were prepared using a TruSeq RNA Sample Prep Kit according to the manufacturer’s manual (Illumina, Inc., San Diego, CA, USA). The poly-A containing mRNA molecules were purified from 1 μg of total RNA from each sample using poly-T oligo-attached magnetic beads. The mRNA was fragmented into approximately 200 bp inserts by sonication. The first-strand cDNA of mRNA fragments was synthesized using reverse transcriptase and random hexamer primers, and the second-strand cDNA was then synthesized using DNA Polymerase I and RNase H. The cDNA fragment then underwent an end processing step, which included the addition of a single “A” base, and then ligation of adapters. The products were then purified and enriched by PCR to amplify the DNA in the library. Size and quantity were confirmed using the Agilent 2200 Tapestation system with High Sensitivity D1000 Tape. After qPCR validation using the KAPA library quantification kit (KAPA Biosystems, Cape Town, South Africa), the libraries were subjected to paired-end sequencing with a 100 bp read length using the Illumina HiSeq 2500 platform, yielding on average 74.2 million reads per library.

### 4.3. De Novo Assembly and Sequence Annotation

High quality reads were obtained by removing adaptor, low-quality sequences, and those that contained a high content of unknown bases (N’s). De novo transcriptome assembly was performed using Trinity [26], an RNA-seq assembler based on the use of a de Bruijin graph. After assembly, we used the TGI Clustering Tool (TGICL) (Available online: http://www.tigr.org/tdb/tgi/software/) to obtain distinct sequences. The resulting assembled transcripts, known as “unigenes”, were analyzed by the TransDecoder tool to predict candidate coding regions within the transcript sequences (Available online: https://transdecoder.github.io/). The unigenes were subjected to searches against the NCBI protein NR (Available online: ftp://ftp.ncbi.nlm.nih.gov/blast/db/), *Arabidopsis thaliana* TAIR10 (Available online: https://phytozome.jgi.doe.gov/), and Clusters of Orthologous Groups (COG, Available online: http://www.ncbi.nlm.nih.gov/COG/) databases, in addition to BLASTX (Available online: http://blast.ncbi.nlm.nih.gov), employing a cutoff *e*-value of 1.0 × 10^−5^. Estimation of transcript abundance was performed using the RSEM package (Available online: http://deweylab.github.io/RSEM/), and the number of reads for each unigene was transformed into FPKM (fragments per kilobase million). Based on the quantification results, the differential expression between the samples was analyzed using the R package [27], with a statistical significance of *p* < 0.01 and ≥1.5-fold change.

### 4.4. Phylogenetic Tree Analysis

The protein sequences were aligned with the CLUSTALW program and the phylogenetic tree was constructed using the MEGA6 software [28] based on the Neighbor-joining method and bootstrapping for 1000 replicates. Accession numbers of genes used in this analysis are listed in Table S3.

### 4.5. Cloning of PtCAS1 and PtCAS2

In order to obtain the full-length cDNA corresponding to the two *PtCAS* genes under the same PCR condition reported by Jin et al. [15], forward and reverse primers were designed as described in Table S4. The fragments were separated by agarose gel electrophoresis, purified using a Wizard PCR Preps Kit (Promega, Madison, WI, USA), and then subcloned into the pYES2.1 TOPO vector (Thermo Fisher Scientific, Waltham, MA, USA). Sequences for the *PtCAS1* and *PtCAS2* genes have been deposited into the GenBank database with accession numbers EU275203 and EU275205, respectively.

### 4.6. Heterologous Expression of PtCAS1 and PtCAS2 Genes in Yeast Mutant

After confirmation of the cDNA sequences of pYES-PtCAS1 and pYES-PtCAS2, the plasmids, including pYES2 (as control), were used for yeast transformation. The transformants were cultured in 10 mL synthetic complete medium without uracil, containing 20 mg/L ergosterol, 13 mg/L hemin, and 5 g/L Tween 80, at 30 °C and 220 rpm. After induction with 2% galactose, the cells were collected and re-suspended in the same volume of 0.1 M potassium phosphate buffer with the same supplements except ergosterol and Tween 80, and incubated for one day at 30 °C. Then, the cells from two flasks were collected into one tube, refluxed with 5 mL 20% KOH and 50% EtOH, and extracted three times with the same volume of *n*-hexane. The extracts were concentrated under a stream of nitrogen gas and re-suspended in 400 μL of CHCl_3_. Lastly, a 100 µL-aliquot was re-concentrated and the trimethylsilylated into pyridine with 50 μL of *N*,*O*-Bis(trimethylsilyl)trifluoroacetamide for 30 min at 70 °C.

### 4.7. Identification of Products from Two CASs

GC-MS analysis was performed under the same conditions as described by Wang et al. [29]. A 1 μL aliquot of the trimethylsilylated solution was analyzed by a 7890N gas chromatography (Agilent Technologies) equipped with a mass spectrometric detector (5973 inert MS, Agilent Technologies) and an HP-5 capillary column (Agilent Technologies; length 30 m, i.d. 250 μm, 0.25 μm, film thickness). Injection condition was at 50 °C for 2 min. The column temperature program was as follows: 40 °C/min ramp to 200 °C, held at 200 °C for 2 min, followed by an increase to 320 °C at a rate of 3 °C/min, and held at 320 °C for 30 min. Cycloartenol was identified by comparison with the standard (purchased from Chromadex, Irvine, CA, USA).

### 4.8. Real-Time RT-PCR Analysis

Total RNA was extracted from the tissue of one-year-old *P. grandiflorum* garden plants. First-strand cDNA was synthesized using the AMV (Avian Myeloblastosis Virus) reverse transcriptase (Promega) and 2 μg of total RNA. SYBR Green Master Mix (Bio-Rad, ‎Hercules, CA, USA.) was used for quantification with the CFX96TM Real-Time System (Bio-Rad) and gene-specific primers (Table S4). PCR conditions were as follows: initial denaturation at 95 °C for 3 min, followed by 40 cycles of amplification for 15 s at 95 °C, for 15 s at 58 °C, and for 30 s at 72 °C. After completing the reactions, the threshold cycle (*C*_t_) value for each reaction was obtained, and the differences were calculated using the ΔΔ*C*_t_ method, and the β-actin gene (GenBank accession no. JK517508) was used as an internal control. The fold change in transcript levels for each gene (considered for qRT-PCR) is presented as the mean and standard error of three independent experiments.

## 5. Conclusions

We generated transcriptome data from 5-week-old seedling treated with MeJA for one day by sequencing on Illumina platform. In the data, a total of 11 unigenes related to the *OSC* gene were detected by gene annotation. Based on sequencing data of two putative *CAS* unigenes, full-length cDNAs of *PtCAS1* and *PtCAS2* genes were isolated and analyzed by a heterologous expression system in yeast cells. Results of GC-MS analysis indicated that two *PtCAS* genes produced cycloartenol as a sole product. However, roles of the two *PtCAS* genes in relation to phytosterol and steroidal triterpenoid biosynthesis remain unclear, despite having elucidated different expression patterns of each in different tissues. Thus, it will be necessary to further evaluate and differentiate functions of the genes in relation to their roles in controlling phytosterol biosynthesis, by overexpression or silencing them. Results may provide us with a greater understanding of the regulatory mechanisms underlying phytosterol biosynthesis and physiological functions of the two *CAS* genes of this plant.

## Figures and Tables

**Table 1:** Summary of *P. tenuifolia* sequencing and assembly. MeJa = methyl jasmonate.

Dataset Name	Control	MeJA (24 h)
No. raw reads	71,835,350	76,653,282
No. clean reads	66,602,004	71,347,274
No. of unigenes	129,035	
Average length of unigenes	1065 bp	
Unigenes expressed in each sample (No.)	121,987	102,869

**Table 2:** Simple sequence repeats (SSR) among different nucleotide types found in the transcriptome of *P. tenuifolia*.

Different Types of SSRs	Number	Proportion (%)	Type No.	Total Length (bp)	Average Length (bp)
Dinucleotide	574	29.4	12	102,317	15
Trinucleotide	1208	61.9	60	216,304	13
Tetranucleotide	112	5.7	72	20,302	17
Pentanucleotide	25	1.3	21	4439	21
Hexanucleotide	31	1.6	31	5559	25

**Table 3:** Differential expression of uingenes of SQS (squalene synthase), SQE (squalene epoxidase), and OSC (oxidosqualene cyclase) candidate genes involved in the biosynthesis of phytosterol and triterpenoid backbone of *P. tenuifolia*. LFC = log fold change; FPKM = fragments per kilobase Million; CON = control.

Enzyme	GeneID	Description	Identity (%)	*e*-Value	LFC	FPKM	
						CON	MeJA
SQS	TBIU012384	squalene synthase (*Polygala tenuifolia*)	99	0	3.31	6.8	47.19
SQE	TBIU001989	squalene epoxidase (*Astragalus membranaceus*)	83	2.0 × 10^−66^	0.765	2.84	3.37
	TBIU001990	squalene monooxygenase (*Theobroma cacao*)	88	2.0 × 10^−83^	13.8	0	17.77
	TBIU020398	PREDICTED: squalene epoxidase 3-like (*Tarenaya hassleriana*)	86	4.0 × 10^−41^	−2.08	1.6	0.26
	TBIU062165	Squalene monooxygenase, putative (*Ricinus communis*)	88	3.0 × 10^−14^	1.82	1.63	4.03
	TBIU068277	PREDICTED: squalene monooxygenase-like (*Eucalyptus grandis*)	88	3.0 × 10^−71^	−1.02	8.19	2.83
OSC	TBIU017624	β-amyrin synthase (*Polygala tenuifolia*)	100	0	4.39	7.69	112.6
	TBIU030037	cycloartenol synthase1 (*Polygala tenuifolia*)	99	3.0 × 10^−102^	1.03	11.05	15.74
	TBIU030038	cycloartenol synthase (*Glycine max*)	91	5.0 × 10^−12^	0.473	2.15	2.09
	TBIU030039	cycloartenol synthase (*Betula platyphylla*)	81	1.0 × 10^−71^	0.013	6.32	4.46
	TBIU030040	PREDICTED : cycloartenol synthase (*Ziziphus jujube*)	80	0	0.699	2.45	2.78
	TBIU030041	cycloartenol synthase1 (*Polygala tenuifolia*)	87	0	0.932	9.48	12.63
	TBIU030042	PREDICTED : cycloartenol synthase (*Ziziphus jujube*)	81	0	0.043	18.15	13.06
	TBIU030043	cycloartenol synthase1 (*Polygala tenuifolia*)	87	0	0.368	1.75	1.58
	TBIU030044	cycloartenol synthase1 (*Polygala tenuifolia*)	99	5.0 × 10^−51^	1.12	9.07	13.84
	TBIU106220	β-amyrin synthase (*Polygala tenuifolia*)	100	1.0 × 10^−14^	2.44	3.47	13.21
	TBIU119757	β-amyrin synthase (*Polygala tenuifolia*)	99	9.0 × 10^−56^	2.81	3.24	15.97

## Supplementary Materials

Supplementary materials can be found at http://www.mdpi.com/1422-0067/18/11/2426/s1.

## Abbreviations

DEUG	Differential Expression of Unigenes
LFC	Log Fold Change
FPKM	Fragments Per Kilobase Million
OSC	Oxidosqualene Cyclase
MeJA	Methyl Jasmonate
CAS	Cycloartenol Synthase
bAS	β-Amyrin Synthase
