# Supplementary material for: Two Cycloartenol Synthases for Phytosterol Biosynthesis in Polygala tenuifolia Willd
Source: Int J Mol Sci. 2017 Nov 15;18(11):2426. doi: 10.3390/ijms18112426 (PMC5713394; doi:10.3390/ijms18112426)
Supplement: Supplementary file 1 [file ijms-18-02426-s001.zip › Table S1 and S2. Accession data for the OSCs..pdf]

Supplemental Table S1. Accession data for the OSCs used in the phylogenetic analyses.

Accession No.	Gene name	Function[Species]
At4g15340	PEN1	Arabidiol synthase [*Arabidopsis thaliana*]
At4g15370	PEN2	Baruol synthase [*A. thaliana*]
At5g36150	PEN3	Tirucalla-7,24-dien-3-ol synthase [*A. thaliana*]
At5g48010	PEN4	Thalianol synthase [*A. thaliana*]
At5g42600	PEN5	Marneral synthase [*A. thaliana*]
At1g78500	PEN6	Bauerenol synthase [*A. thaliana*]
At1g78970	LUP1	Multifunctional triterpene synthase [*A. thaliana*]
At1g78960	LUP2	Multifunctional triterpene synthase [*A. thaliana*]
At1g78955	LUP3	Multifunctional triterpene synthase [*A. thaliana*]
At1g78950	LUP4	β-amyrin synthase [*A. thaliana*]
At1g66960	LUP5	Multifunctional triterpene synthase [*A. thaliana*]
At3g45130	LSS1	Lanosterol synthase [*A. thaliana*]
At2g07050	CAS1	Cycloartenol synthase[*A. thaliana*]
AB263204	RsM2	Multifunctional triterpene synthase [*Rhizophora stylosa*]
AB257507	KcMS	Multifunctional triterpene synthase [*Kandelia candel*]
AB289586	BgLUS	Lupeol synthase [*Bruguiera gymnorhiza*]
FJ032006	MdOSC1	Multifunctional triterpene synthase [*Malus* × *domestica*]
FJ032008	MdOSC3	Multifunctional triterpene synthase [*Malus* × *domestica*]
KT383435	MdOSC4	Multifunctional triterpene synthase [*Malus* × *domestica*]
KT383436	MdOSC5	Multifunctional triterpene synthase [*Malus* × *domestica*]
AB058643	LcIMS1	Isomultiflorenol synthase [*Luffa cylindrical*]
FJ013228	NsbAS	β-amyrin synthase [*Nigella sativa*]
HM623869	KdGLS	Multifunctional triterpene synthase [*Kanlanchoe daigremontiana*]
HM623870	KdFRS	Multifunctional triterpene synthase [*K. daigremontiana*]
HM623871	KdLUS	Multifunctional triterpene synthase [*K. daigremontiana*]
AB609123	SHS1	Shionone synthase [*Aster tataricus*]
AY836006	AsOXA1	β-amyrin synthase [*Aster sedifolius*]
FJ790411	GsAS1	β-amyrin synthase [*Gentiana straminea*]
KJ467352	GsAS2	β-amyrin synthase [*G. straminea*]
AB037203	GgbAS	β-amyrin synthase [*Glycyrrhiza glabra*]
AB181244	LjAMY1	β-amyrin synthase [*Lotus japonicus*]
AF478455	LjAMY2	Multifunctional triterpene synthase [*L. japonicus*]
AJ430607	MtAMY1	β-amyrin synthase [*Medicago truncatula*]
AB03802	PSY	β-amyrin synthase [*Pisum sativum*]

EF107623	PtBS	β-amyrin synthase [*Polygala tenuifolia*]
AB034803	PSM	Multifunctional triterpene synthase [*Pisum sativum*]
DQ915167	SvBS	β-amyrin synthase [*Saponaria vaccaria*]
AB206469	EtAS	β-amyrin synthase [*Euphorbia tirucalli*]
AB289585	BgbAS	β-amyrin synthase [*B. gymnorhiza*]
AB263203	RsM1	Multifunctional triterpene synthase [*R. stylosa*]
AB055512	BPY	β-amyrin synthase [*Betula platyphylla*]
HM623868	KdTAS	Multifunctional triterpene synthase [*K. daigremontiana*]
AB009030	PNY1	β-amyrin synthase [*Panax ginseng*]
HM219225	AeAS	β-amyrin synthase [*Aralia elata*]
HQ266579	SlTTS1	β-amyrin synthase [*Solanum lycopersicum*]
HQ266580	SlTTS2	Multifunctional triterpene synthase [*S. lycopersicum*]
AB265170	PNA	Dammarenediol-II synthase [*P. ginseng*]
JN991165	CrAS	Multifunctional triterpene synthase [*Catharanthus roseus*]
AB009029	PNX	Cycloartenol synthase [*P. ginseng*]
AB055510	BPX	Cycloartenol synthase [*B. platyphylla*]
AB025968	GgCAS1	Cycloartenol synthase [*G. glabra*]
D89619	PSX	Cycloartenol synthase [*P. sativum*]
HM623872	KdCAS	Cycloartenol synthase [*K. daigremontiana*]
AK121211	AK121211	Cycloartenol synthase [*O. sativa*]
AK070534	AK070534	Unnamed protein [*O. sativa*]
AB116238	CPQ	Cucurbitadienol synthase [*Cucurbita pepo*]
HQ128567	SgCbQ	Cucurbitadienol synthase [*Siraitia grosvenorii*]
AB009031	PNZ	Lanosterol synthase [*P. ginseng*]
AK066327	AK066327	Pakeol synthase [*O. sativa*]
AB025343	QEW	Lupeol synthase [*Olea europaea*]
AB025345	TRW	Lupeol synthase [*T. officinale*]
AB055511	BPW	Lupeol synthase [*B. platyphylla*]
KX147270	MiFRS	Friedelin synthase [*Maytenus ilicifolia*]
KX147271	MiCAS1	Cycloartenol synthase [*M. ilicifolia*]
KM111167	IaAS1	Multifunctional triterpene synthase [*Ilex asprella var. asprella*]
KM111168	IaAS2	Multifunctional triterpene synthase [*I. asprella var. asprella*]
JQ728553	WsOSCBS	β-amyrin synthase [*Withania somnifera*]
JQ728552	WsOSCLS	Lupeol synthase [*W. somnifera*]
HM037907	WsOSCCS	Cycloartenol synthase [*W. somnifera*]

Supplemental Table S2. Oligonucleotide primers used in this study

	Primers	Sequence (5' to 3')
Cloning	PtCAS1-F	TCATGTGGAGGCTGAAGATCGCAAAC
	PtCAS1-R	TTAGCATGCCTGCAATACACGAGTA
	PtCAS2-F	TCATGTGGAAACTTAAGATCGGAGCA
	PtCAS2-R	TTAAAGCGAGTGTAACACTTTCGAT
qRT-PCR	PtCAS1-F	ATACCGTACTCGTGTATTGCAG
	PtCAS1-R	CAGTAACAGTTGACAAGGCGA
	PtCAS2-F	CCAATATGGGCATTGGGTGAA
	PtCAS2-R	ATGTCAAACGACAACATCCCAA
	Actin-F	GATGACATGGAAAAGATTTGGCATC
	Actin-R	AAGGATGGCATGAGGGAGGGCGTAA
